# Glucocorticoids and endothelial function in inflammatory diseases: focus on rheumatoid arthritis

**DOI:** 10.1186/s13075-016-1157-0

**Published:** 2016-11-05

**Authors:** Frank Verhoeven, Clément Prati, Katy Maguin-Gaté, Daniel Wendling, Céline Demougeot

**Affiliations:** 1 EA 4267 FDE, FHU INCREASE, Université Bourgogne Franche-Comté, F-25030 Besancon, France; 2 Service de Rhumatologie, CHRU Besançon, F-25030 Besançon, France; 3 EA 4266, Université Bourgogne Franche-Comté, F-25030 Besancon, France

**Keywords:** Glucocorticoids, Endothelial function, Rheumatoid arthritis

## Abstract

Rheumatoid arthritis (RA) is the most common systemic autoimmune disease characterized by articular and extra-articular manifestations involving cardiovascular (CV) diseases. RA increases the CV mortality by up to 50 % compared with the global population and CV disease is the leading cause of death in patients with RA. There is growing evidence that RA favors accelerated atherogenesis secondary to endothelial dysfunction (ED) that occurs early in the course of the disease. ED is a functional and reversible alteration of endothelial cells, leading to a shift of the actions of the endothelium towards reduced vasodilation, proinflammatory state, proliferative and prothrombotic properties. The mechanistic links between RA and ED have not been fully explained, but growing evidence suggests a role for traditional CV factors, auto-antibodies, genetic factors, oxidative stress, inflammation and iatrogenic interventions such as glucocorticoids (GCs) use. GCs have been used in RA for several decades. Whilst their deleterious CV side effects were described in the 1950s, their effect on CV risk associated with inflammatory arthritis remains subject for debate. GC might induce negative effects on endothelial function, via a direct effect on endothelium or via increasing CV risk factors. Conversely, they might actually improve endothelial function by decreasing systemic and/or vascular inflammation. The present review summarizes the available data on the impact of GCs on endothelial function, both in normal and inflammatory conditions, with a special focus on RA patients.

## Background

Rheumatoid arthritis (RA) is the most common systemic autoimmune disease characterized by reduced life expectancy ranging between 3 and 18 years compared with the general population [1]. The relative increase in risk of myocardial infarction and stroke is 68 % and 41 %, respectively [1]. The leading cause of excess mortality in patients with RA is cardiovascular disease (CV) triggered by accelerated atherogenesis [1]. The CV risk in RA is roughly twice that of the general population and was found to be comparable to the risk in diabetes [2]. Most of the evidence suggests that “traditional” risk factors account for only part of the excess CV risk [1]. Other mechanisms, probably specific to RA, or increased by RA, such as high-grade inflammation, are likely to play a role. Many, if not all, of these risk factors appear to affect CV health through changes in the endothelium. Indeed, in a wide range of CV diseases endothelial activation and dysfunction precedes and initiates atherosclerosis [3]. Endothelial dysfunction has prognostic value for CV events and its correction is associated with reduced CV risk [3]. Evidence from clinical studies has shown that endothelial dysfunction (ED) is present in established RA [4] and is impaired by genetic factors, like the presence HLA-DRB1*04 shared epitope alleles [5].

A woman with severe RA was successfully treated with glucocorticoids (GCs) for the first time in 1948, yet the majority of patients still use GCs in combination with disease-modifying antirheumatic drugs (DMARDs), and exceptionally alone, despite significant evolution in the treatment of RA. The link between GC and CV disease has been known since the 1950s when Adlersberg et al. demonstrated that the effect of cortisone on lipids was involved in premature development of atherosclerosis [6]. Since then, evidence obtained from the global population has shown that therapeutic doses of oral GC (≥7.5 mg/day) are associated with increased CV disease and all-cause mortality [7].

GCs are responsible for insulin resistance, modification of the lipid profile and hypertension [8]. However, the effects of GCs on CV risk in inflammatory disease seem more complex. While GCs might increase CV risk by increasing CV risk factors, it might also reduce CV risk by decreasing the systemic and/or vascular inflammation. In giant cell arteritis, which is a large-vessel systemic vasculitis associated with high inflammatory burden, endothelial function was found to be impaired in individuals with active disease. Nevertheless, corticosteroids improved the endothelial function following suppression of the inflammation [9].

The effects of GCs on CV risk in RA have been addressed in recent reviews [10, 11] and have yielded variable results. GCs were found to increase CV and/or mortality risk, particularly with increasing doses [10, 11], or to have no effect or an uncertain effect [12]. As preserving endothelial function appears to be a major goal in mitigating CV risk in RA [4], the aim of the present review is to provide the available data on the effects of GCs on endothelial function, with a special focus on RA.

## Normal endothelial function

The endothelium is a monocellular layer that plays a role as a physical barrier, being the interface between the blood and the vessel wall. However, the endothelium is not just a static tissue but also a crucial homeostatic organ for the regulation of vascular tone and structure. It senses mechanical stimuli, such as pressure and shear stress, and chemical stimuli, such as hormones and locally secreted vasoactive substances [3]. In response to these stimuli the endothelium releases factors that regulate vasomotor function, inflammatory processes, cell growth, and hemostasis. Since the importance of endothelium was first recognized by its capacity to release factors that modulate vascular tone [3], these factors are classically classified as endothelium-derived relaxing factors (EDRF) including nitric oxide (NO), prostacyclin (PGI_2_) and endothelium-derived hyperpolarizing factor (EDHF), and endothelium-derived contracting factors (EDCF) including angiotensin-II, endothelin-1 and vasoconstrictor prostanoids.

## Vascular expression of GC receptors

The synthetic GCs used in RA are agonists of glucocorticoid receptors (GR) and to a lesser extent of mineralocorticoid receptors (MR), which are both members of the nuclear receptor superfamily of ligand-activated transcription factors [13]. The main GR responsible for the therapeutic effects of GC is GRα which is expressed throughout the body, whereas MR is expressed in relatively few tissues [14]. Thus, the cellular response to GCs depends on whether the tissue expresses MR and/or GR. In the vascular system, GR and MR are expressed by intact arteries, cultured vascular smooth muscle cells (VSMC), and endothelial cells [8]. Thus, the conditions exist for a direct modulation of endothelial function by GCs.

## Endothelial dysfunction in RA

Endothelial dysfunction (ED) is a widely used term to describe any form of abnormal functional and reversible alteration of endothelial cells, leading to an abnormal or inappropriate response to physiological stimuli and to a shift of the actions of the endothelium toward reduced vasodilation, proinflammatory state, and proliferative and prothrombotic properties [3, 15]. ED is an important early event in the pathogenesis of atherosclerosis, contributing to plaque initiation and progression [15]. The role of ED as the *sine qua non* condition for atherosclerosis development makes it an early indicator of disease at a stage that may allow for effective risk factor modification or pharmacologic intervention prior to the development of atherosclerosis.

Many techniques are available for assessing endothelial function in humans [16]. The most commonly used is the non-invasive method called flow-mediated dilation (FMD), which evaluates macrovascular endothelial function. FMD relies on the measurement by ultrasound of the vasodilatory response of the brachial artery to post-ischemic hyperemia. The limitations of this method are that it is a technically demanding technique and the duration of ischemia, which is variable. Microvascular endothelial function can be measured by invasive methods such as the forearm blood flow (FBF) technique, or non-invasive techniques such as laser Doppler skin flowmetry or digital pulse amplitude tonometry (PAT), but this new method need to be validated. The limitation of the FBF is its invasive nature and its duration.

ED in patients with RA was first described in 2002 [17]. This study reported the impairment in the brachial artery responsiveness to acetylcholine, assessed by FBF in patients with early disease. Since then, numerous publications have confirmed the presence of ED in RA [4, 16]. ED was described both in the macrovasculature and in the microvasculature [18] in early RA [19] and in well-established disease [20] in patients with low [21] or high disease activity [22]. ED is present in patients with established RA, who in most cases do not have classic cardiovascular risk factors [6]. Thus, a role of inflammation in the development of ED is highly suspected but the channels through which rheumatic inflammation leads to ED are not completely clear. Data about endothelial mechanisms involved in ED have been provided by studies on animal models of RA. As reported in a recent review [23], RA-associated ED is secondary to decreased NO availability, decreased endothelial NOS expression/activity, uncoupling of endothelial NOS, increased arginase activity, excess of superoxide anion production, impaired EDHF production, increased synthesis of prostanoids and increased angiotensin II production.

## Direct effects of GCs on endothelial cells and function

Evidence from in vitro and ex vivo models suggests that GCs are able to directly modulate endothelial function. However, their effects seem different depending on whether they are used in healthy conditions or in conditions associated with inflammation.

### Effects of GCs on endothelial function in the physiological condition

**Fig. 1:**
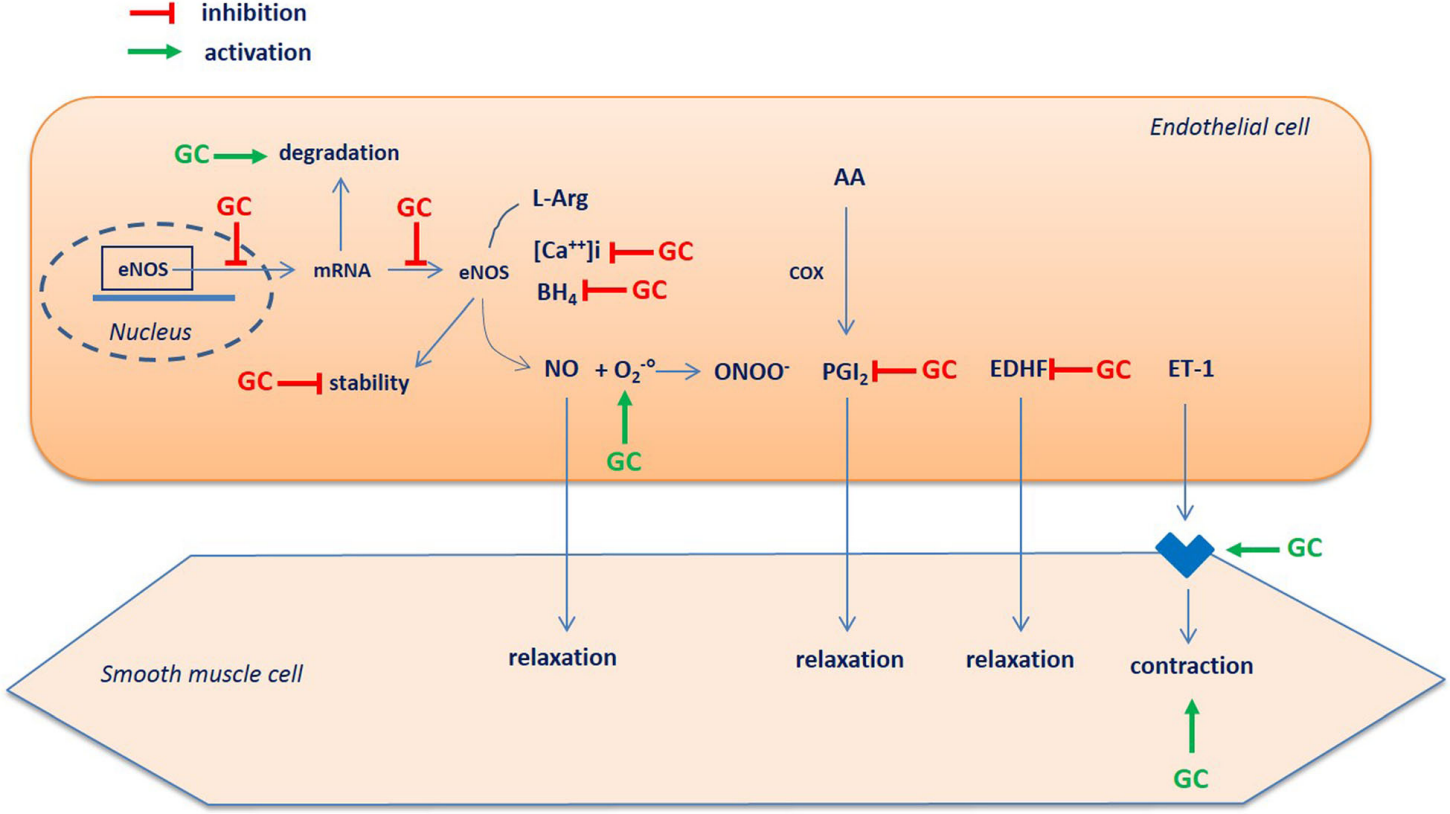
Effects of glucocorticoid (*GC*) on endothelial function in normal conditions. *eNOS* endothelial nitric oxide synthase, *L-Arg* L-Arginin, *BH4* tetrahydrobiopterin, *O*_*2*_^*-*^ superoxide anion, *ONOO* ^*-*^ peroxynitrite anion, *EDHF* endothelium derived hyperpolarizing factor, ET-1 endothelin-1, *AA* arachidonic acid, *PGI*_*2*_ prostacyclin

GC treatment in animals leads to impaired endothelial function [24, 25]. As regards the mechanisms involved (Fig. 1), decreased vascular availability in NO, the major mediator of endothelial function produced by the vascular endothelial NO synthase (eNOS), has been demonstrated [25], which is secondary to decreased eNOS activity [26], eNOS expression [25], eNOS gene transcription [24], increased degradation of eNOS mRNA [27], decreased eNOS protein stability [24], inhibition of calcium mobilization in endothelial cells [26] or reduction of tetrahydrobiopterin levels, a cofactor required for eNOS enzyme activity [28]. Besides decreasing eNOS activity/expression, GCs have beem found to reduce vascular NO bioavailability by increasing reactive oxygen species (ROS) production. Exposure of cultured endothelial cells to dexamethasone increases ROS production by NAD(P)H oxidase and xanthine oxidase, decreases NO production and increases the production of the cytotoxic peroxynitrites (ONOO^-^) [24, 29].

Other studies demonstrate that NO is not the sole EDRF modulated by GCs. In a model of rats treated with a GR agonist, the treatment blunted aortic prostacyclin production [30]. Regarding EDHF, in a rat model of prenatal dexamethasone exposure, GC was responsible for an imbalance between EDHF and EDCF production, to the detriment of EDHF in the aorta [31]. GCs are also responsible for increased sensibility to endothelium-derived vasoconstrictors, such as endothelin-1 [25, 32].

In light of the above data obtained from tissue culture studies or studies in experimental animals, one would expect GCs to hamper endothelial function in humans. Surprisingly, the few studies exploring the impact of GCs on endothelium-dependent vasodilation in humans did not fully confirm this assumption. In 30 patients receiving 2 mg/kg/day of prednisolone orally for 4 weeks, endothelial function assessed by FBF was unchanged by the treatment [33]. Likewise, acute variations in plasma GC concentrations (after an intravenous (i.v.) bolus of hydrocortisone) did not impair endothelial function measured by FBF in healthy men [34]. In 16 healthy male volunteers, an acute dose of oral prednisolone (single 50 mg dose) did not change the response to acetylcholine assessed by forearm venous occlusion plethysmography as compared to placebo [35]. Conversely, the response to hyperemia measured by FBF in GC-treated patients was decreased as compared to the response before GC therapy. The administration of vitamin C almost normalized the blood flood response. Another study also identified significant attenuation of acetylcholine-induced vasodilation after 5 days but not after 2 days of hydrocortisone treatment [36]. These results could imply that although GC can produce detrimental defects in endothelial function, these are exerted over a period of time rather than acutely. In this latter study, the authors emphasized that the mineralocorticoid effects of hydrocortisone that may lead to ED could have biased the interpretation of the data.

### Effects of GCs on endothelial function in conditions associated with inflammation

**Fig. 2:**
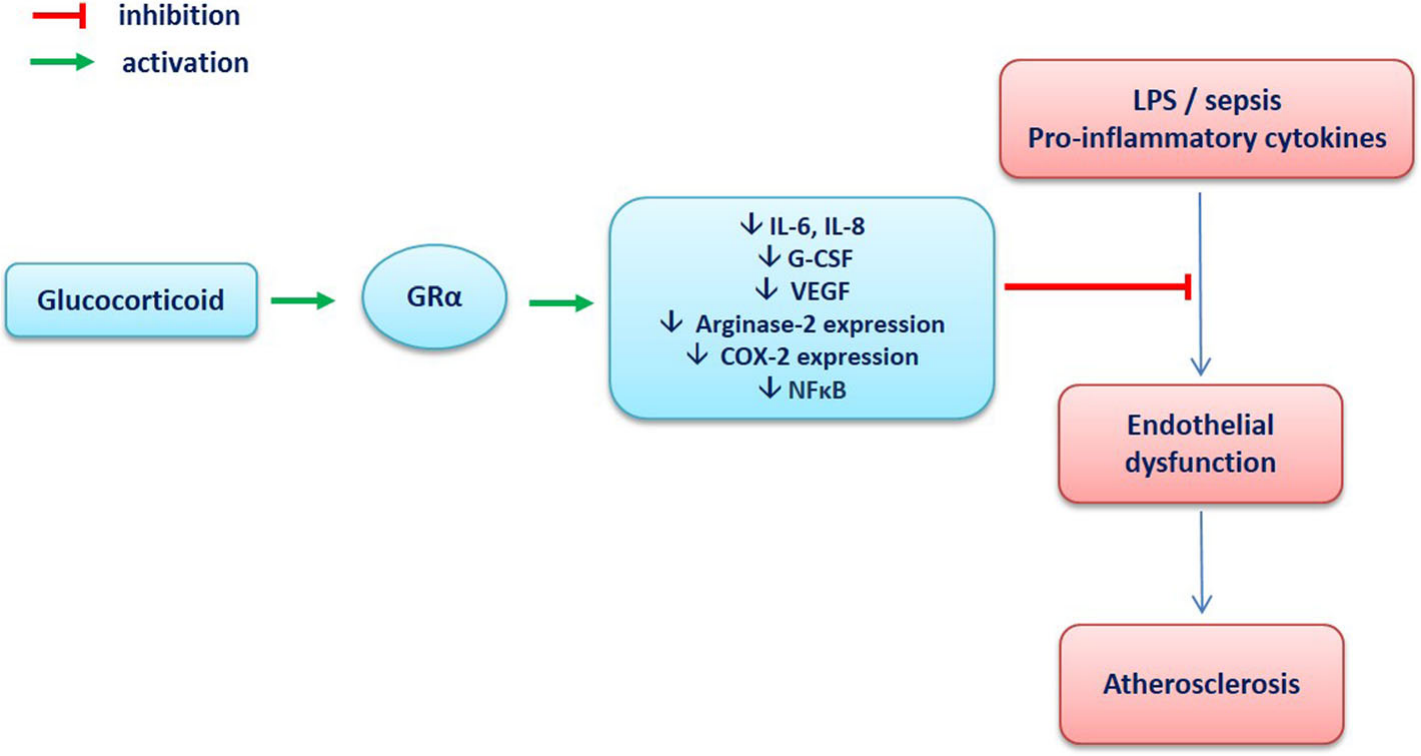
Effects of glucocorticoid (GC) on endothelial function in inflammatory conditions. *GRα* glucocorticoid receptor α, *G-CSF* granulocyte-colony stimulating factor, *VEGF* vascular endothelial growth factor, *COX-2* cyclooxygenase 2, *LPS* lipopolysaccharides

The role of the GCs on endothelial function in inflammatory conditions seems different compared to physiological conditions (Fig. 2). Compelling evidence argues for a beneficial vascular effect of GCs in high-grade inflammation associated with septic shock and this effect is mediated by the activation of the endothelial GC receptor [37, 38]. Interestingly, these positive effects of GCs seem also present in conditions associated with low-grade systemic inflammation, such as atherosclerosis [8]. Atherosclerotic mice lacking the endothelial GC receptor exhibit more severe atherosclerotic lesions than control mice, indicating that the endothelial GC receptor is important for the inhibition of atherosclerotic progression [39]. In a model of atherosclerotic mice (APOE*3-Leiden.CETP mice), both transient and continuous corticosterone treatment decreased the total atherosclerotic lesion area [40]. Similar results were found after chronic administration of dexamethasone in a model of cholesterol-fed rabbits [41]. The mechanisms involved in the beneficial effects of GC on endothelial cells in inflammatory conditions are likely due to a decrease in endothelial expression of cytokines (IL-6, IL-8), G-CSF, VEGF, endothelin-1, NFκB [42], arginase 2 [43] and COX-2 [44].

## Effects of GC on endothelial function in RA

The forementioned animal data suggest that GCs might exert beneficial effects on endothelial function in a disease with a high level of inflammation, such as RA.

**Fig. 3:**
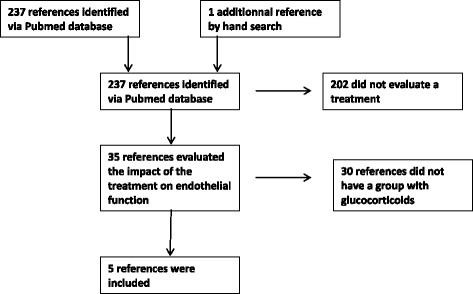
Flow chart

We performed a review of the literature (Fig. 3) using the Pubmed database with the following keywords: “rheumatoid arthritis” AND “endothelial function” AND “treatment”. We completed with a hand search. The search was restricted to studies in English and on humans older than 18 years. We identified 238 studies and 35 were selected after reading the titles: finally, after examination 5 studies were relevant for analysis (Table 1).

**Table 1:** Effects of GCs on endothelial function in RA

	Country of the study	Number of patients	Disease duration (years)	Disease activity (DAS 28)	Treatment	GC treatment duration	Control group	Evaluation criteria	Effect on endothelial function	Newcastle-Ottawa scale
Hafström et al. [45]	Sweden	13	<2	3.0 ± 1.3	Prednisolone 7.5 mg/day (p.o.) + DMARD	5 years	DMARD alone	FMD	3.44 % ± 2.08 vs 3.74 % ± 2.9 (NS)	8
Ikonomidis et al. [46]	Greece	19	10	5.3 ± 1.1	Prednisolone upper dose 5 mg/day (p.o.) + DMARD	30 days	Compared to baseline	FMD	5.0 % ± 1.9 vs 4.3 % ± 1.6 (NS)	9
Foster et al. [47]	England	3	10.7	-	Methylprednisolone 500 mg (i.v.)	Single dose	Compared to baseline	FMD	3.3 % vs 3.1 % (NS)	4
Veselinovic et al. [48]	Serbia	52	5.72	3.69 ± 0.84	Prednisolone 7.5 mg/day (p.o.) + DMARD	At least 1 year	RA patients without GC	FMD	9.16 % ± 7.03 vs 12.6 % ± 5.49 *P* = 0.005	7
Radhakutty et al. [49]	Australia	18	-	-	Prednisolone 6 mg/day (p.o.)	7 days	RA patients taking prednisolone for 6 months (4–10 mg/day)	PAT	No effect	7

*GC* glucocorticoid, *RA* rheumatoid arthritis, *DAS28* disease activity score in 28 joints, *DMARD* disease-modifying antirheumatic drug, *i.v*. intravenous, *p.o.* orally, *FMD* flow-mediated dilation, *FBF* forearm blood flow, *PAT* peripheral arterial tonometry, *NS* not significant

In a first study [45], 67 patients with early active RA were recruited (at the time of prescription of the first DMARD). Among them 34 patients received 7.5 mg/day of prednisolone in addition to the DMARD and 33 received only the DMARD. In the group treated with prednisolone + DMARDs, only 13 patients were exclusively treated without treatment change over 5 years. After 5 years of treatment, endothelial function was measured by FMD of the brachial artery and atherosclerotic plaques were attested by ultrasonography. There was no difference in FMD and atherosclerotic plaques between the “DMARD alone” compared to the “DMARD + prednisolone” group. However, the DMARD + prednisolone group had hypertension and hypercholesterolemia more frequently compared to the DMARD alone group. The authors concluded that a low dose of GCs did not influence endothelial function and atherosclerosis in patients with RA. The major limitation of this study was the use of a DMARD that might exert is own effect on endothelial function.

Ikonomidis et al. [46] compared the effect of anakinra (an IL-1 receptor antagonist) on FMD of the brachial artery to treatment with a non-biologic DMARD (methotrexate, leflunomide or hydroxychloroquine) and to an upper dose of 5 mg/day of prednisolone over 30 days. While patients treated with anakinra exhibited significantly improved FMD, patients treated with prednisolone had lower FMD than control subjects both before and after treatment (*P* < 0.05), but without improvement of FMD after the treatment. The main limitation was that this study was not designed to evaluate the effects of GCs on endothelial function. The dose of prednisolone was unclear and the group size was small (19 patients).

Forster et al. [47] investigated the impact of GCs on endothelial function of the macrocirculation (assessed by FMD) and the microcirculation (assessed by FBF) in patients (mean age 58.5 years; disease duration 10.7 years) with chronic RA. In this study, a subgroup of 29 patients required an additional antirheumatic treatment (anti-TNFα or methotrexate or an i.v. bolus of 500 mg methylprednisolone). In this subgroup, endothelial function was measured before the beginning of the new treatment, and 2 and 4 weeks later. Endothelial function (micro and macro) was not modified by GC treatment. Thus, infusion of a high dose of methylprednisone did not worsen endothelial function (but did not improve it) after 4 weeks. The limitations were the lack of history on the patients with RA and the possible co-prescription of methotrexate or anti-TNFα as a confounding factor.

Veselinovic et al. [48] investigated the correlation between intima-media thickness and FMD in 52 patients (mean age 52.46 years, disease duration 5.72 years) with RA treated with one or two DMARDs (methotrexate ± hydroxychloroquine). Among these patients, 33 (63 %) were treated with a low dose of corticosteroids (7.5 mg/day of prednisone) continuously for a minimum of 1 year. FMD was significantly lower in RA as compared to controls (*P* = 0.005). Patients with RA taking low doses of GCs had significantly enhanced FMD compared to those who did not use corticosteroids. The main limitation of this study was the use of a non-biologic DMARD by every patient.

Very recently, Radhakutty et al. [49] aimed at comparing acute vs chronic treatment with GCs on post-prandial endothelial function in patients with RA. Microvascular endothelial function (assessed by PAT) was measured before and after a standardized meal, in 18 patients with RA (mean age 66 years), who were taking continuous oral prednisolone 4–10 mg/day for at least 6 months (chronic treatment) or 18 patients with RA (mean age 64 years) taking a 7-day course of oral prednisolone 6 mg/day (acute treatment). The results showed that there was no difference in post-prandial endothelial function between chronic and acute GC treatment. The main study limitations were the lack of information on RA duration or severity, the use of DMARDs in some patients, the small group size and the lack of a control group.

## Conclusion

Despite their well-documented adverse effects, GCs are still included in the American College of Rheumatology (ACR)/European League Against Rheumtism (EULAR) recommendations for the management of RA. Given the seminal role of ED as the precursor of CV diseases, it is important to determine and understand whether GCs are likely to influence endothelial function in patients with RA. Data from experimental models highlight the Janus face of GCs: they can exert deleterious effects on endothelial function when applied in “normal” cells/animals but they also seem protective under inflammatory conditions. In patients with RA, data are scarce and controversial. While most studies showed no deterioration of endothelial function after a treatment with a low dose or a bolus of GCs, one study reported improvement in endothelial function after 1 year of treatment with a low dose of a GC [48]. The effects of GCs are likely independent on disease duration [49]. Thus, despite the classical assumption that GCs are responsible for deterioration in endothelial function, no available data in the literature demonstrate such an effect in RA. However, the present review pointed out the great heterogeneity in the design of available studies (various stages of RA disease, various dosages of GCs or treatment durations) which makes the results of these studies inconclusive. Therefore, there is an urgent need to perform clinical trials specifically designed to define the best GC strategy to prevent CV risk in RA.

## Abbreviations

COX: cyclooxygenase
CV: cardiovascular
DMARDs: disease-modifying antirheumatic drugs
ED: endothelial dysfunction
EDCF: endothelium-derived contracting factors
EDHF: endothelium-derived hyperpolarizing factor
EDRF: endothelium-derived relaxing factor
eNOS: endothelial nitric oxide synthase
FBF: forearm blood flow
FMD: flow mediated dilation
GCs: glucocorticoids
G-CSF: granulocyte-colony stimulating factor
GR: glucocorticoid receptor
IL: interleukin
i.v.: intravenous(ly)
MR: mineralocorticoid receptor
mRNA: messenger RNA
NADPH: reduced nicotinamide adenine dinucleotide phosphate
NFκB: nuclear factor-kappa B
NO: nitric oxide
NOS: nitric oxide synthase
PAT: pulse amplitude tonometry
PGI_2_: prostacyclin, RA, rheumatoid arthritis
ROS: reactive oxygen species
TNFα: tumor necrosis factor α
VEGF: vascular endothelial growth factor
